# Acute but not chronic metabolic acidosis potentiates the acetylcholine-induced reduction in blood pressure: an endothelium-dependent effect

**DOI:** 10.1590/1414-431X20155007

**Published:** 2015-12-04

**Authors:** A.C. Celotto, L.G. Ferreira, V.K. Capellini, A.A.S. Albuquerque, A.J. Rodrigues, P.R.B. Evora

**Affiliations:** Departamento de Cirurgia e Anatomia, Faculdade de Medicina de Ribeirão Preto, Universidade de São Paulo, Ribeirão Preto, SP, Brasil

**Keywords:** Acid-base balance, Metabolic acidosis, Hemodynamic failure, Endothelium, Nitric oxide

## Abstract

Metabolic acidosis has profound effects on vascular tone. This study investigated the in vivo effects of acute metabolic acidosis (AMA) and chronic metabolic acidosis (CMA) on hemodynamic parameters and endothelial function. CMA was induced by ad libitum intake of 1% NH4Cl for 7 days, and AMA was induced by a 3-h infusion of 6 M NH4Cl (1 mL/kg, diluted 1:10). Phenylephrine (Phe) and acetylcholine (Ach) dose-response curves were performed by venous infusion with simultaneous venous and arterial blood pressure monitoring. Plasma nitrite/nitrate (NOx) was measured by chemiluminescence. The CMA group had a blood pH of 7.15±0.03, which was associated with reduced bicarbonate (13.8±0.98 mmol/L) and no change in the partial pressure of arterial carbon dioxide (PaCO2). The AMA group had a pH of 7.20±0.01, which was associated with decreases in bicarbonate (10.8±0.54 mmol/L) and PaCO2 (47.8±2.54 to 23.2±0.74 mmHg) and accompanied by hyperventilation. Phe or ACh infusion did not affect arterial or venous blood pressure in the CMA group. However, the ACh infusion decreased the arterial blood pressure (ΔBP: -28.0±2.35 mm Hg [AMA] to -4.5±2.89 mmHg [control]) in the AMA group. Plasma NOx was normal after CMA but increased after AMA (25.3±0.88 to 31.3±0.54 μM). These results indicate that AMA, but not CMA, potentiated the Ach-induced decrease in blood pressure and led to an increase in plasma NOx, reinforcing the effect of pH imbalance on vascular tone and blood pressure control.

## Introduction

Severe acidosis can affect cardiovascular function and decrease the cardiac output, arterial blood pressure, and hepatic and renal blood flow (1). Additionally, pH can modulate the vascular tone (2). Most research has shown that acidosis induces relaxation mediated by nitric oxide (NO/cGMP-dependent) and prostacyclin (PGI2/cAMP-dependent) and hyperpolarizes the cell membrane (3,4). The pH of the blood and extracellular fluids is generally maintained at around 7.4, but some acute and chronic disorders such as hypoxia, diabetes mellitus, diarrhea, and renal disorders can disrupt acid-base homeostasis, leading to systemic acidemia (5), a common problem in intensive care units.

The scientific literature contains many reports on the acid-base balance and endothelial function, but these concepts are not yet clear, mainly with respect to conductance arteries. Additionally, many recent works have presented conflicting data on the relationship between metabolic acidosis and endothelial function (6-10). The effects of pH and its mechanisms of action are expected to vary among vessel types, duration of acidosis (acute versus chronic), and severity of acidosis (11). However, most *in vivo* studies have assessed the effects of acidosis induced by ventilatory changes in the pCO_2_; the vascular effects of metabolic acidosis remain poorly investigated. Thus, this study was carried out to evaluate the effects of acute and chronic acidosis on hemodynamic and biochemical parameters in rabbits. The *in vivo* vascular response to the vasoconstrictor phenylephrine (Phe) and the nitric oxide (NO/cGMP-dependent) vasodilator acetylcholine (Ach) during acute metabolic acidosis (AMA) and chronic metabolic acidosis (CMA) was assessed indirectly by measuring blood pressure. Metabolic acidosis was assessed in the context of the clinical state (e.g., sepsis or circulatory shock).

## Material and Methods

### Animals

Male New Zealand rabbits weighing 2.5-3.0 kg were obtained from the Central Animal Facility of the Campus de Ribeirão Preto, Universidade de São Paulo (USP). The animals were kept at an ambient temperature (22°C-25°C) and controlled light/dark cycle (12:12 h). The animals had free access to water and food. The experimental protocols involving the animals were approved by the Ethics Committee on Animal Experimentation, Faculdade de Medicina de Ribeirão Preto, USP, São Paulo, SP, Brazil.

### Protocol for induction of AMA and CMA

AMA was induced by continuous infusion of 6 M NH_4_Cl (1 mL/kg, diluted 1:10) at an average flow of 10 mL/h for 3 h, while control animals received only saline (11). CMA was induced by oral administration (*ad libitum*) of an NH_4_Cl 1%+0.5% glucose solution for 7 days (12). The controls received 0.5% glucose only. The proposed protocol aimed to achieve a pH close to 7.1. Eight animals were used in each experiment.

### Experimental procedures

Anesthesia was induced by intramuscular injection of 10 mg/kg of xylazine and 50 mg/kg of ketamine in one of the hind legs. A slow (about 10-min) infusion of one-third the initial dose through the ear vein was performed every 30 min to maintain analgesia and sedation. The animals received an intravenous infusion of 10 mL·kg^-1^·h^-1^ of 0.9% sodium chloride to compensate for sensitivity loss during the procedure. The animals were kept under spontaneous ventilation with an oxygen catheter. After anesthesia, the left femoral vein and femoral artery were cannulated with a polyethylene catheter. An MP 100 system (Biopac Systems, Inc., USA) was employed to monitor the mean arterial pressure and mean venous pressure and to record the electrocardiogram. At the end of the experiment, the animals were sacrificed by exsanguination.

After catheter placement, the operator waited 20 min to ensure stabilization of parameters and collection of the basal data. The pre-drug data were collected immediately before infusion of Ach and Phe. The *in vivo* study of vascular function was performed by intravenous (dorsal ear vein) infusion of ACh (total dose, 15 µg/kg) and Phe (total dose, 15 µg/kg) for 2 min each (13-15). A dose-response curve of each agonist was performed as follows: solution 1 (5 µg/kg of agonist) infused over 2 min; solution 2 (10 µg/kg of agonist) infused over 2 min. Arterial and venous pressures were evaluated during the 4-min infusion of ACh and Phe. The dose-response curve was followed by collection of the post-drug data.

### Analysis of biochemical parameters

The following gases and electrolytes were analyzed (GEM Premier 3000; Instrumentation Laboratory, USA) from heparinized arterial blood collected from the femoral artery: pH, arterial bicarbonate (HCO_3_^−^), hematocrit, hemoglobin, ion serum calcium, sodium, potassium, partial pressure of arterial carbon dioxide (PaCO_2_), and oxygen saturation. Blood samples were collected every 30 min during AMA induction.

### Plasma nitrite/nitrate measurement

For plasma nitrite/nitrate (NO_x_) analysis, 1.5 mL venous blood samples were collected from the femoral vein. Each blood sample was placed in a standard collection tube containing 0.08 mL of heparin (BD Vacutainer K2EDTA; Becton, Dickinson and Company, USA), transported on ice (-20°C), and centrifuged at 3000g for 10 min at 4°C. The plasma was deproteinated and stored at -70°C for later analysis of plasma NO_x_ by NO/ozone chemiluminescence using the Sievers¯ Nitric Oxide Analyzer 280 (GE Analytical Instruments, USA).

### Statistical analysis

Statistical analyses were performed using analysis of variance (ANOVA), the Bonferroni *post-test*, or Student’s *t*-test by GraphPad Prism, Version 4.0 (GraphPad Software Corporation, USA) according to the type of data. Statistical significance was set at P<0.05.

## Results

**Figure 1:**
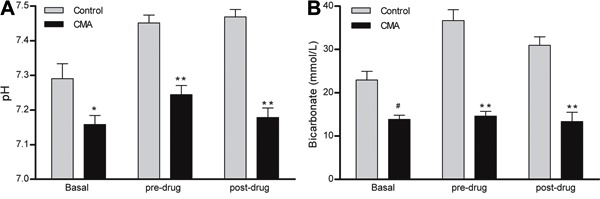
pH (*A*) and bicarbonate measurements (*B*) in response to chronic metabolic acidosis (CMA). Basal, pre-drug (before dose-response curves), and post-drug (after dose-response curves) data are reported as means±SE. *P<0.05 and **P<0.0001, compared to control (Student’s *t*-test; n=8).

**Figure 2:**
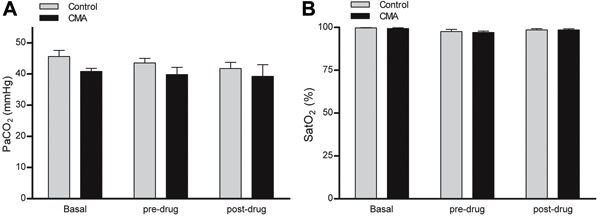
*A*, Partial pressure of arterial carbon dioxide (PaCO_2_) and *B*, oxygen saturation (SatO_2_) measurements in response to chronic metabolic acidosis (CMA). Basal, pre-drug (before dose-response curves), and post-drug (after dose-response curves) data are reported as means±SE (P>0.05, Student’s *t*-test; n=8).

**Figure 3:**
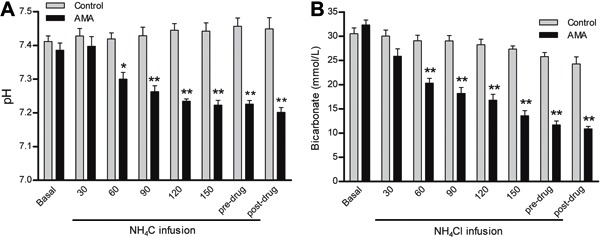
*A*, pH and *B*, bicarbonate measurements in response to acute metabolic acidosis (AMA). Basal, pre-drug (before dose-response curves), and post-drug (after dose-response curves) data are reported as means±SE. *P<0.01 and **P<0.001 compared to control (two-way repeated-measures ANOVA and Bonferroni *post-test*; n=8).

**Figure 4:**
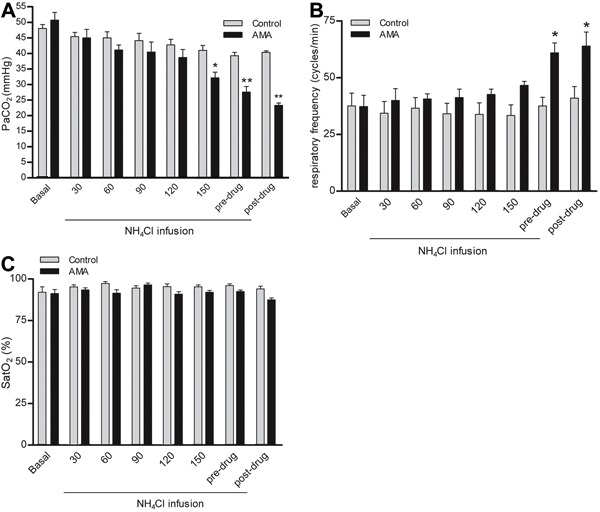
*A*, Partial pressure of arterial carbon dioxide (PaCO_2_), *B*, respiratory frequency, and *C*, oxygen saturation (SatO_2_) in response to acute metabolic acidosis (AMA). Basal, pre-drug (before dose-response curves), and post-drug (after dose-response curves) data are reported as means±SE. *P<0.01 compared to control (two-way repeated-measures ANOVA and Bonferroni *post-test*; n=8).

After 1 week of CMA, the pH was 7.15±0.03, which was associated with a blood HCO_3_^−^ of 13.8±0.98 mmol/L (Figure 1A and B). These animals did not present any changes in PaCO_2_ or oxygen saturation (Figure 2A and B). In the AMA model, after 3 h of NH_4_Cl infusion, the pH was 7.22±0.01 and was associated with a blood HCO_3_^−^ of 11.7±0.82 mmol/L (Figure 3A and B). The PaCO_2_ decreased to 27.6±1.8 mmHg and was accompanied by simultaneous hyperventilation, but no changes in oxygen saturation were observed (Figure 4A, B, and C). Thus, the protocols were successful in creating AMA and CMA in the rabbits.

**Figure 5:**
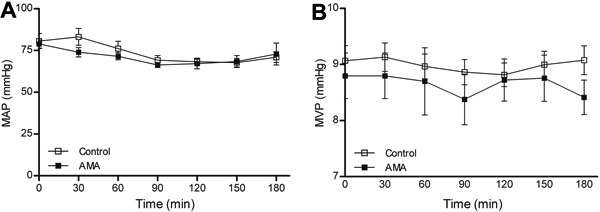
*A*, Mean arterial pressure (MAP) and *B*, mean venous pressure (MVP) during induction of acute metabolic acidosis (AMA). Data are reported as means±SE (P>0.05, two-way repeated-measures ANOVA and Bonferroni *post-test*; n=8).

No differences in cardiac frequency or cardiac output were found in the CMA or AMA groups (data not shown), and the animals in the AMA group did not show any changes in arterial or venous blood pressures during the induced acidosis (Figure 5A and B).

**Figure 6:**
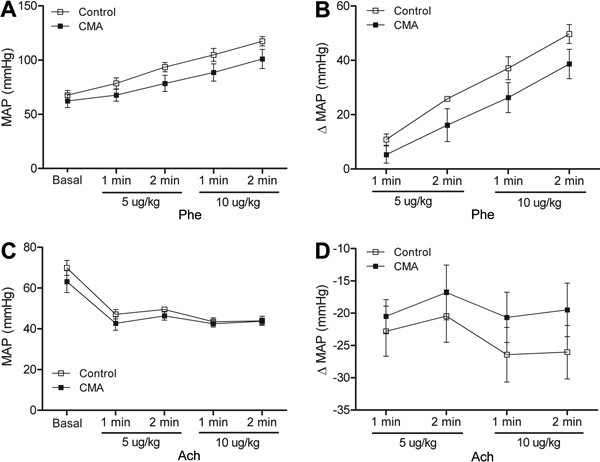
Results from chronic metabolic acidosis (CMA). *A*, Mean arterial pressure (MAP), *B*, MAP variation (Δ) by phenylephrine; *C*, MAP, and *D*, MAP variation (Δ) in response to acetylcoline. Data are reported as means±SE (P>0.05, two-way repeated-measures ANOVA and Bonferroni *post-test*; n=8).

**Figure 7:**
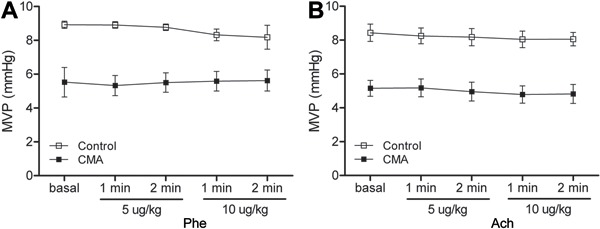
Results from chronic metabolic acidosis (CMA). *A*, Mean venous pressure (MVP) in response to phenylephrine and *B*, mean venous pressure in response to acetylcholine. Results are reported as means±SE (P>0.05, two-way repeated-measures ANOVA and Bonferroni *post-test*; n=8).

**Figure 8:**
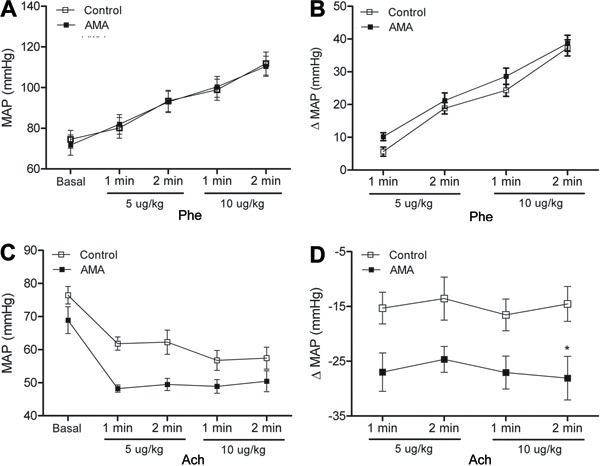
Results from acute metabolic acidosis (AMA). *A*, Mean arterial pressure (MAP) and *B*, MAP variation (Δ) in response to pheylephrine; *C*, MAP, and *D*, MAP variation (Δ) in response to acetylcholine. Results are reported as means±SE. *P<0.05 compared to control (two-way repeated-measures ANOVA and Bonferroni *post-test*; n=8).

**Figure 9:**
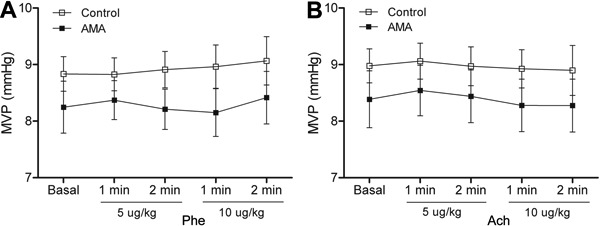
*A*, Mean venous pressure (MVP) in response to phenylephrine (*A*) and acetylcholine (*B*) during acute metabolic acidosis (AMA). Results are reported as means±SE (P>0.05, two-way repeated-measures ANOVA and Bonferroni *post-test*; n=8).

Vascular reactivity (measured indirectly by blood pressure variation) in the CMA group showed that the increase in arterial blood pressure in response to Phe (Figure 6A and B) was similar in both groups. Likewise, no differences in the Ach curves were observed between the groups (Figure 6C and D). During the Phe and ACh curves, the venous blood pressure remained unchanged (Figure 7A and B). When the dose-response curves were performed after acute acidosis, the blood pressure increase by Phe was the same for the control and AMA groups (Figure 8A and B). However, the decrease in arterial blood pressure by ACh infusion was higher in the AMA than control group (ΔBP: -28.0±2.35 mmHg [AMA] to -14.5±2.89 mmHg [control]) (Figure 8C and D). During the Phe and ACh curves, the venous blood pressure remained unchanged (Figure 9A and B).

**Figure 10:**
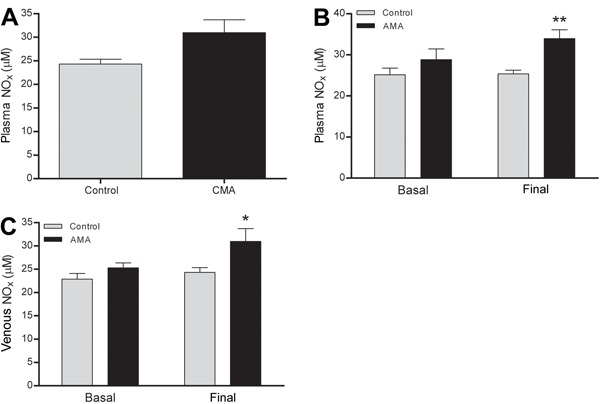
Plasma nitrite/nitrate (NO_x_) levels. *A*, Arterial plasma NO_x_ in response to chronic metabolic acidosis (CMA), *B*, arterial plasma NO_x_ in response to acute metabolic acidosis (AMA), and *C*, venous plasma NO_x_ in response to AMA. Results are reported as means±SE *P<0.05 and **P<0.01 compared to control (Student’s *t*-test; n=8).

The plasma NO_x_ remained unchanged in response to CMA (Figure 10A) but increased in the arterial and venous blood in response to AMA (Figure 10B and C). Plasma NO_x_ measurement in the CMA group was performed only at the end of the experiments. Among the biochemical blood parameters (sodium, potassium, calcium, hemoglobin, hematocrit, lactate, and glucose), only potassium was higher in the AMA than in the control group (Table 1), and glucose was lower in the CMA group at the beginning of the experiment than in the control group (Table 2).

**Table:**
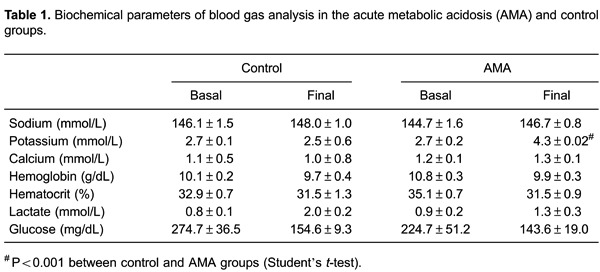

**Table:**
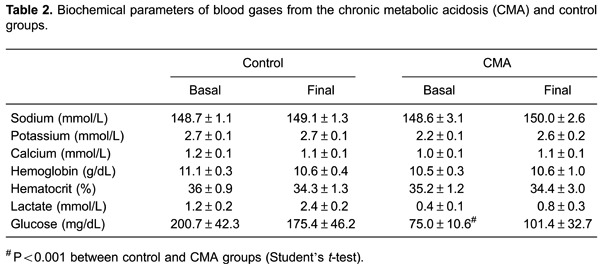

## Discussion

Metabolic acidosis can promote extracellular and intracellular pH imbalances, which affect cellular function. Treatment should be directed at improving homeostasis for both (16). An understanding of all body responses under a condition of acidosis is necessary to identify the most appropriate treatment.

The experimental methods used to induce AMA and CMA in this study were effective because the pH range achieved was 7.15 to 7.22 with a bicarbonate of 11.7 to 13.8 mmol/L in both acidosis models. The induction of CMA through 1 week of NH_4_Cl administration was carried out easily, and only 1 of the 10 treated animals did not develop acidosis and 1 died, probably due to dehydration. Even with the addition of glucose, the NH_4_Cl used for CMA treatment likely reduced the water palatability for the animals, reducing the ingested volume compared with the control group. This phenomenon could have been responsible for the dehydration in some animals as observed by clinical examination and the reduction in mean venous pressure in the others. Animals that developed CMA showed increased serum urea and creatinine concentrations (data not shown). Studies that showed increased serum urea concentrations in a rat model of NH_4_Cl-induced CMA attributed the elevated urea to an increase in protein catabolism and activation of urea synthesis to eliminate excess NH_4_^+^ (17,18). On the other hand, the increase in creatinine may have resulted from a reduction in the renal filtration caused by low fluid intake, leading to a change in renal function.

Conversely, optimizing the AMA protocol was quite laborious because no other successful protocol had been published in rabbits. Previously attempted protocols in other animal species, such as HCl infusion, either led to animal death or were ineffective in reducing the pH. Creation of an AMA model has been time-consuming and costly; however, we achieved success with an adaptation from a model performed in calves using NH_4_Cl (11).

The animals with AMA only showed an increase in urea (data not shown), perhaps because of the activation of urea synthesis to eliminate excess NH_4_^+^ (18). The increase in potassium, observed in this group may have resulted in potassium efflux from the cell in an attempt to maintain electroneutrality before the entrance of excess H^+^ ions into the cell (19). The increase in potassium levels did not promote changes in the heart rate, and cardiac output was also maintained under conditions of AMA, showing that the model used to induce AMA did not impair cardiac contractility. The PaCO_2_ decreased at the end of the experiment in animals with AMA. This observation was accompanied by an increase in respiratory rate, indicating respiratory compensation associated with acidosis. If PaCO_2_ is not appropriately depressed, the severity of the acidemia and intracellular acidosis at a given serum HCO_3_^−^ concentration would be greater (20).

To assess its severity, metabolic acidosis has been divided into three forms based on the systemic arterial blood pH: mild (pH of 7.30-7.36; associated with HCO_3_^−^ of >20 mmol/L), moderate (pH of 7.20-7.29; associated with HCO_3_^−^ of 10-19 mmol/L), and severe (pH of <7.20; associated with HCO_3_^−^ of <10 mmol/L). Although this categorization is arbitrary, it could help clinicians to make decisions about the requirement for and type of treatment (21). Both acidosis conditions achieved in this study could be classified as moderate acidosis.

In the CMA studies, administration of a vasodilator (Ach) or a vasoconstrictor (Phe) promoted an equal decrease and increase in the blood pressure in the control or CMA animals. Along with the hemodynamic data, the plasma NO_x_ levels showed that CMA per se did not affect vascular function in the study protocol or that the animals had undergone some adaptation process to maintain the integrity of vascular function.

AMA is characterized by many different effects including a decrease in cardiac contractility and cardiac output, arterial vasodilatation, and a predisposition to cardiac arrhythmias, contributing to sudden death (22-24).

The results of our *in vivo* AMA experiment showed that relaxation induced by Ach was increased under conditions of acidosis. Additionally, the plasma NO_x_ level was increased in acidosis. These results suggest that AMA enhances the vasodilatory effect of ACh, mediated by increased NO_x_ release. Pedoto et al. (25) showed that metabolic acidosis may increase inducible nitric oxide synthase and that this may lead to vasodilation and shock, as occurs under conditions of sepsis. The effect of acidosis over nitric oxide production was also shown in our previous studies, but in this case, endothelial nitric oxide synthase is involved (16). Decades ago, nitric oxide synthase was shown to be activated by acidosis and plasma NO_x_ was shown to become more stable in an acidic medium (26,27). Corroborating our results, Kellum et al. (24) found that moderate acidosis promotes hypotension mediated by increases in plasma NO_x_, while severe acidosis promotes only hypotension without changes in plasma NO_x_.

In contrast to our results of Phe infusion, Huang et al. (28) showed that the responsiveness to both endogenous and infused catecholamines is attenuated when accompanied by acidosis.

The effect of metabolic acidosis on hemodynamics is varied and complex. Acidosis has been shown to stimulate vasopressin, adrenocorticotropic hormone, and aldosterone in experimental animal models and may therefore increase the blood pressure; however, hypotension may also occur. Most studies of acidosis and vascular function have investigated the sensitivity of inducible nitric oxide synthase to decreases in pH. In the present study, however, we propose an understanding of acidosis exclusively without the involvement of any disease. We observed that acute metabolic acidosis per se can increase plasma NO_x_, leading to hypotension and shock independently of the primary disease. Given the important effects of acute metabolic acidosis on clinical outcomes, more intensive studies of the pathogenesis of acidosis are necessary to find novel and efficient treatment methods. Future studies should explore the mechanisms by which metabolic acidosis promotes increases in plasma NO_x_ and why this occurs only in the acute setting.

Metabolic acidosis is common in seriously ill patients and is associated with increased morbidity and mortality because of its depressive effects on cardiovascular function and hemodynamic instability. In the present study, we showed that AMA increased Ach responsiveness by increases in plasma NO_x_. An improved understanding of the time-dependent events that occur during AMA could result in the development of a structured approach to treatment at various time points of the disorder.
